# Evaluation of immunosuppressive function of regulatory T cells using a novel *in vitro* cytotoxicity assay

**DOI:** 10.1186/2045-3701-4-51

**Published:** 2014-09-01

**Authors:** Linyi Zhang, Jean N Manirarora, Cheng-Hong Wei

**Affiliations:** Gene Transfer and Immunogenicity Branch, Division of Cellular and Gene Therapies, Office of Cellular, Tissue, and Gene Therapies, FDA, Center for Biologics Evaluation and Research, Bethesda, Maryland USA

**Keywords:** Regulatory T cells, Immunoregulation, Antigen specific, CD8 T cells, NOD

## Abstract

Naturally occurring regulatory T cells (Tregs) play a pivotal role in the maintenance of self-tolerance due to their intrinsic immunosuppressive activity. Currently, a number of human clinical trials are being conducted to investigate the roles of Tregs in treating various immune-mediated disorders. Traditionally, the suppressive activity of Tregs is measured using either a thymidine incorporation assay, which is a radioactive assay; or CFSE based flow cytometry assay, which requires a relatively large number of cells. Consequently, there is an increasing need to develop novel alternative bioassays that can characterize various aspects of the immunosuppressive function of Tregs *in vitro*. In this study, using murine clonal CD8^+^ T cells specific for an islet antigen as responder T cells, we first established a novel, sensitive and quantitative *in vitro* luminescence based cell viability assay to measure cytotoxicity. Then we used this assay to measure if Tregs could inhibit the cytotoxicity of CD8 effector T cells. This assay does not involve the use of radioisotopes and only needs relatively low number of Tregs. Since normally Tregs only constitute 5-10% of peripheral CD4^+^ T cells, this advantage is noteworthy compared with other methods. With the assay we developed, we demonstrated that regulatory T cells (Tregs) could inhibit the antigen-specific killing of an adherent target cell monolayer by the CD8^+^ cytotoxic T cells. We observed more inhibition when Tregs and CD8 killer T cells were incubated during the *in vitro* activation (stimulation) stage of the cytotoxic T lymphocytes (CTL) than when they were added later at the start of the effector phase. Interestingly, Tregs from B6 mice demonstrated higher suppression of CD8^+^ T cell killing than Tregs from NOD mice. Moreover, IL-2/anti-IL-2 mAb complexes induced expansion of Tregs *in vivo*, as well as enhancing the Treg’s suppressive activity per cell.

Therefore, this novel non-radioactive, luminescence based cytotoxicity assay mediated by clonal islet antigen-specific CD8 T cells can be used to measure, characterize, and quantitate the immunosuppressive activity of natural Tregs, representing a useful approach to characterize the functions of Tregs in the setting of autoimmune diseases and to elucidate the mechanisms for Treg cell-mediated immunoregulation.

**Electronic supplementary material:**

The online version of this article (doi:10.1186/2045-3701-4-51) contains supplementary material, which is available to authorized users.

## Introduction

Naturally occurring regulatory T cells (Tregs), are CD4^+^CD25^+^Foxp3^+^ T cells capable of inhibiting the function of effector T cells to regulate immunological homeostasis [1]. Based on their tissue origin and differentiation, Tregs can be divided into thymus-derived natural Tregs (nTreg) and peripherally induced Tregs (iTregs).

There is a large amount of evidence for the role of CD4^+^CD25^+^ Tregs in the maintenance of self-tolerance. If Treg cells are artificially depleted, the result is lymphoproliferative disease resulting in death [2]. In Scurfy mice the Foxp3 gene is mutated, inactivating their Tregs and resulting in severe autoimmunity that affects multiple organs [3]. In humans, similarly, mutation and loss of function of Foxp3 leads to a decrease in regulatory T cell number and function, leading to the severe multi-organ autoimmune disease IPEX (immune dysregulation, polyendocrinopathy, enteropathy, and X-linked inheritance) and death [4, 5]. Based on these observations, several studies have demonstrated that adoptive transfer of Tregs can be a promising immunotherapy of autoimmune diseases in mice. For instance, it has been reported that adoptively transferred Tregs can ameliorate the development of autoimmune diseases in several murine models, including type 1 diabetes [6, 7], EAE (experimental autoimmune encephalomyelitis) [8, 9], colitis [10, 11], arthritis [12], and lupus [13].

In regards to autoimmune type 1 diabetes (T1D) in which insulin-producing beta cells are destroyed by auto-reactive T cells, many studies have investigated the role of CD4^+^CD25^+^ Treg cells in regulating the onset and progression of the disease [14, 15]. These studies have demonstrated that supplementation of the endogenous CD4^+^CD25^+^ Treg cell population provides resistance to the disease, whereas depletion of Treg cells accelerates T1D progression, showing that CD4^+^Foxp3^+^ Treg cells are critical in the control of T1D in mouse models.

As reviewed by Buckner [16], Treg cell defects can lead to autoimmune diseases. Defects include: decreased number of Treg cells due to poor proliferation, survival or stability; defects in Treg cell function (such as failed contact-dependent suppression, decreased secretion of soluble factors such as IL-10, TGF-β); and resistance of effector T cells to the suppression by Treg cells. In non-obese diabetic (NOD) mice (a model of spontaneous type 1 diabetes), Treg cell numbers and Foxp3 expression decrease with the age of the animals [17], Treg function also declines with aging of the NOD mice [18], although there were also studies where decrease of Treg frequencies was not discovered [19, 20]. Adoptive transfer of Treg cells can ameliorate disease in NOD mice [6], but over time the pathogenic effector T cells in the pancreatic islets become resistant to suppression [21–23]. All these studies have confirmed the important role of Treg cells in the control of diabetes onset and progression, and that a decrease in CD4^+^Foxp3^+^ Treg cell frequencies or function in NOD mice, constitutes a critical predisposing factor to T1D [15].

In order to examine whether there is a defect or abnormality in Treg function in certain human immune diseases, sensitive and quantitative *in vitro* assays are needed. Various kinds of suppression assays have been developed to measure the suppression of responder T cell function by Tregs. For example, the thymidine incorporation assay has been used most frequently, in which suppression of anti-CD3 mAb stimulated proliferation of CD4^+^CD25^−^ T cells (conventional T cells, Tconv) is measured by [^3^H] thymidine incorporation [24, 25]. The shortcoming of this assay is that it cannot distinguish which specific cell population in the co-culture has incorporated [^3^H] thymidine. Needless to say, a radioactive isotope is used in this assay.

Another commonly used method is the CFSE-based cell proliferation assay using FACS. It is similar to the [^3^H] based assay in that this assay also measures proliferation, but the proliferation of CD4^+^CD25^−^ T cells is measured by the decrease of green fluorescence from CFSE dye when cells divide [26]. The advantages of this method are that it can specifically evaluate the proliferation of the responder T cell population (can be CD4 or CD8 T cell subsets), as well as to examine the number of cell divisions throughout the culture period [27]. However, the limitation of CFSE dilution assays is that they require a larger number of Tregs than [^3^H] thymidine incorporation assay.

Besides these methods, two other methods have also been reported. One is a cytokine production assay, in which the ability of Treg cells to inhibit the production of cytokines by conventional T cells stimulated with anti-CD3 mAb is measured [28]. Another assay is based on the measurement of surface markers, for example, it has been reported that Treg function can be quantified through measuring their suppression of up-regulation of surface markers (such as CD154, CD69) on Tconv following activation [29].

All of these methods have been commonly utilized by different labs and provided very useful information for our understanding of Treg cells. It needs to be noted, however, that in most if not all cases, naïve non-antigen specific T cells were used as the responder population, and anti-CD3 mAb was used for activation. Thus there is a lack of a functional test on regulatory T cells’ function in regulating antigen specific T cells. This issue is particularly relevant for studies aiming at suppressing pathogenic auto-reactive effector T cells, such as T cells specific for self-tissue antigens (for instance, islet antigens, myelin basic protein-derived antigens, thyroid antigens). As documented in the literature, immune rejection and autoimmune responses to islet antigens have been a major hurdle to achieving long term engraftment of islet transplants [30], and even genetically engineered insulin-producing cells cannot escape from this kind of autoimmune attack [31]. Tregs which can suppress this type of autoimmune responses provide an alternative for immunotherapy to improve the long-term survival of islets without depending on long-term immunosuppressive drugs.

To address this specific need, in this study we have developed a novel, sensitive and quantitative luminescence based assay that does not involve the use of radioactive isotope, uses relatively low number of Treg cells, and can be easily modified to a high-throughput format for screening purposes. In addition, islet-antigen specific CD8 T cells were exploited as responder T cells, and antigen-specific lysis of islet cells was used as a readout to measure the inhibitory function of Treg cells.

## Materials and Methods

### Ethics statement

All animal protocols and procedures were approved by the Institutional Animal Care and Use Committees (IACUC) of the Center for Biologics Evaluation and Research (CBER) and performed in animal facilities accredited by the Association for Assessment and Accreditation of Laboratory Animal Care International. All experiments were performed according to institutional guidelines.

### Cell culture

The insulinoma cell line NIT-1 (ATCC#CRL-205) established from the islet beta cells of SV40 large T antigen transgenic NOD mice [32], the fibroblast cell line NIH 3T3(ATCC#CRL-1658), and the BALB/c derived fibroblast cell line BALB/3T12-3 (ATCC# CCL-164) were all purchased from American Type Culture Collection (ATCC) (Manassas, VA) at passage one. NIT-1 cells were cultured in T175 flasks, expanded in RPMI-1640 medium supplemented with 10% FBS, penicillin, streptomycin and L-glutamine, and cultured at 37°C and 5% CO_2_. NIH-3T3 cells and BALB/3T12-3 cells were grown in DMEM and harvested with 0.25% Trypsin (Life Technologies, Grand Island, NY). NIT-1, NIH-3T3, and BALB/3T12-3 were used at passage numbers of P2 – P40. When performing sub-culturing, the cell surface was rinsed twice with 2 ml of cell dissociation buffer (Life Technologies, Grand Island, NY) or Trypsin, and then incubated for approximately 6–8 minutes until cells were detached. Subsequently, 3 ml of cell culture media was added to the flask and cells were split at the desired ratios. During the assay, cell density and viability were determined using the Cellometer®Vision (Nexcelom Bioscience, Lawrence MA). To perform the experiments, the cell density was adjusted to 2000/50 μl in each testing well, equal to 4x10^4^ cells/ml. At this concentration, 50 μl cell suspension was added to the inner 60 wells of a 96-well tissue-culture treated plate for a final density of 2,000 cells/well. The tissue culture treated white plates used in the assay (Corning Costar, Cat. #: 3917) were made of polystyrene with white wall and flat and white bottom.

### Mice

NOD/ShiLtj mice, Nonobese diabetic 8.3 TCR transgenic mice (NOD 8.3) [33] were purchased from Jackson Laboratories (Bar Harbor, Maine) and were maintained in specific pathogen-free conditions. The 8.3 TCR transgenic CD8^+^ T cells specifically recognize the K^d^-restricted IGRP _206–214_ epitope derived from the islet antigen IGRP (islet-specific glucose-6-phosphatase catalytic subunit-related protein) [34].

### Peptide

The peptides IGRP _206–214_ (VYLKTNVFL) and H-2K^d^-binding peptide Tum (KYQAVTTTL) were synthesized and HPLC purified by FDA core facility. Purified IGRP and TUM were diluted to 10 mg/ml with DMSO and stored at −20°C.

### Flow cytometry analysis

Cells were incubated with antibodies at the optimized concentrations for 30 minutes at 4°C. The samples were centrifuged, washed twice with PBS/1% fetal bovine serum, and analyzed using a FACSCanto (Becton Dickinson) flow cytometer. For flow cytometry analysis of regulatory T cells, anti-CD4, anti-CD8, anti-CD25, anti-GITR, anti-CD62L, and anti-Foxp3 antibodies in various fluorochrome combinations were purchased from BD Biosciences (San Diego, CA).

### Isolation and purification of CD4^+^CD25^+^ Tregs

Lymphocytes were obtained from the lymph nodes and spleens of NOD or B6 mice treated with or without IL-2/anti-IL-2 mAb complexes. CD4^+^CD25^+^ Tregs were isolated using a Treg isolation kit (Miltenyi Biotech, Auburn, CA). Briefly, CD4^+^ T cells were first negatively selected on a separation column, then the CD4^+^CD25^+^ and CD4^+^CD25^−^ subsets were purified using PE-anti-CD25 antibody and anti-PE magnetic beads (Miltenyi Biotech, Auburn, CA) according to the manufacturer's instructions. The purity of CD4^+^CD25^+^ regulatory cell was consistently higher than 90%, as confirmed by FACS.

### Cytotoxicity assay as measured by CellTiter-Glo® (CTG) method

The CTG based cytotoxicity assay is designed to determine the number of viable cells left in the culture as a result of cytotoxic T lymphocyte killing of the target cells. The assay involves the luciferase reaction, in which mono-oxygenation of luciferin is catalyzed by the UltraGlo^TM^ recombinant luciferase in the presence of Mg^2+^, molecular oxygen, and ATP stemming from the lysates of viable cells. As a result of the reaction, a luminescent signal defined as a relative luminescent unit (RLU) is generated, which is proportional to the amount of ATP present. The amount of ATP is directly proportional to the number of metabolically active cells. A direct relationship exists between the luminescent signal and the number of viable cells in culture. The lower the number of viable cells associates with the strong killing capacity of effector CD8^+^ T cells.

In the assay, lymphocytes were obtained from the lymph nodes and spleens of NOD 8.3 TCR transgenic mice. The CD8^+^ T cells were isolated using a mouse CD8^+^ T cell isolation kit (Miltenyi Biotech). After isolation, 2x10^6^ CD8^+^ 8.3 T cells were seeded into a 24-well plate, stimulated with anti-CD3/CD28 Ab conjugated beads (Life Technologies, Grand Island, NY) at a bead to cell ratio of 2 to 1 in the presence of 2000 IU/ml of human IL-2 (PeproTech, Rocky Hill, NJ) in complete RPMI 1640 medium (2 ml/well) for 3 days in a CO2 incubator. Then magnetic beads were removed using a DynaMag^TM^-2 Magnet (Life Technologies). Subsequently the *in vitro* activated 8.3 T cells were counted and applied as the effector cytotoxic T cells in the CTG assay to detect their killing capacities over the NIT-1 target cells. To set up an assay plate, 50 μl of NIT-1 cells were seeded into a 96-well white plates at indicated concentration (2,000 cells/well unless specified otherwise), and *in vitro* activated 8.3 T cells were added to each well at the indicated effector cell (8.3 T cells) to target cell (NIT-1 cells) ratio of 5:1. Simultaneously, IGRP peptide was added to each well at a final concentration of 20 μg/ml. After the overnight incubation (12–16 hours), suspension T cells (both 8.3 T cells and Tregs) were removed by three repetitive pipetting and washing with cell culture media. The luminescent signal (relative luminescent unit, RLU) from a 96-well plate was measured by the addition of 200 μl of 50% Cell Titer Glo (Promega, Madison, WI) followed by measurement of luminescent signals using a GloMax multi-detection system (Promega, Madison, WI). The % killing of the NIT-1 cells by 8.3 CD8^+^ T cells was calculated according to the following equation: % Killing = 100% x (RLU of untreated NIT-1 cells – RLU of NIT-1 cells cultured with 8.3 CD8^+^ T cells)/RLU of untreated NIT-1.

### Preparation of murine IL-2/anti-IL-2 mAb complexes

1 μg of murine IL-2 (PeproTech, Cat.#: 212–12) was incubated with 5 μg of anti-mouse IL-2 mAb (eBioscience, Clone: JES6-1, Cat.#: 16-7022-85; San Diego, CA) at 4°C for 15 min to allow the formation of immune complexes. Subsequently, the complexes of murine IL-2/anti-mouse IL-2 mAb were injected into mice via *i.p.* route in 100 μl HBSS to each recipient NOD mouse for 3 days (1 dose/day).

### Testing the immunosuppressive activity of Tregs on CD8^+^ T cell cytotoxicity during the activation phase

NOD mice were treated with IL-2/anti-IL2 mAb complexes (i.p.) on day 0, 1, and 2. On day 5, CD4^+^CD25^+^ Tregs were isolated from the untreated NOD or IL-2/anti-IL-2 mAb complexes treated NOD mice. To investigate the role of Tregs during the activation phase, freshly isolated Tregs and 8.3 CD8 T cells were cultured together in the presence of the anti-CD3/anti-CD28 Ab conjugated Dynabeads and human IL-2 for 3–4 days. On the day of assay (the last day of activation of CD8 T cells), CD8 T cells were separated from Treg cells by negative selection using the mouse Dynabeads CD4 (L3T4) from Invitrogen. The NIT-1 cells were adjusted to 2000/well. The 8.3 CD8 T cells were used at an E/T ratio of 5:1. The 8.3 CD8^+^ T cells and NIT-1 cells plus IGRP peptide were mixed together and incubated overnight. After overnight incubation, 8.3 T cells were excluded by washing three times with cell culture medium while adherent NIT-1 cells remained in the testing wells. Subsequently, the viability of NIT-1 cells was measured by the addition of 200ul of 50% Cell Titer Glo, which reflected the killing capacity of 8.3 T cells over the NIT-1 cells.

### Testing the immunosuppressive activity of Treg on CD8 T Cell Cytotoxicity during the effector phase

NOD or B6 mice were left untreated or treated with IL-2/anti-IL2 mAb complexes (i.p.) on day 0, 1, and 2. On day 2, TCR transgenic 8.3 CD8^+^ T cells were isolated from the spleen/lymph nodes using CD8 T cell isolation kit (Miltenyi) and activated in the presence of hIL-2 and CD3/CD28 Dynabeads for 3 days. On day 5, CD8^+^ 8.3 T cells were separated from the beads with a DynaMag^TM^-2 Magnet (Life Technologies), counted and resuspended in media. On the same day, CD4^+^CD25^+^ Tregs were isolated from the spleen/lymph nodes of NOD or B6 mice treated with or without the IL-2/anti-IL-2 mAb complexes, counted, and then mixed with 8.3 T cells and NIT-1 cells plus exogenous IGRP peptide. A fixed number of NIT-1 cells (2000 cells/well) and fixed E/T ratio of 5:1 (5 8.3 CD8^+^ T cells to 1 NIT-1 cell) were used, and titrated number of Tregs was added to obtain different ratios of Tregs to 8.3 T cells. After 12 to 16 hours of incubation, the viability of NIT-1 cells was measured by the addition of 200ul of 50% Cell Titer Glo, which reflected the killing capacity of 8.3 T cells over the NIT-1 cells. The immunosuppressive activity of Tregs was calculated as % inhibition of killing.

### CFSE Immunosuppression assay

Spleens and lymph nodes were isolated from Thy1.1 NOD 8.3 TCR transgenic mice, then CD8^+^ T cells were purified using the mouse CD8^+^ T cell isolation kit (Miltenyi Biotech). 8.3 CD8^+^ T cells were labelled with CFSE (InvitroGen, Carlsbad, CA) according to the manufacturer’s instructions, added to 24 well plates (Becton Dickinson Labware, Franklin Lakes, NJ) (2 x 10^6^ cells/well). Subsequently anti-CD3/CD28 beads were added to each well at the bead to T cell ratio of 2 to 1, and then 2x10^6^ of CD4^+^CD25^+^ Tregs isolated from untreated or IL-2/anti-IL-2 mAb complexes treated NOD mice were added to the 24-wells plates where 8.3 CD8^+^ T cells were seeded. The cells were kept in RPMI 1640 complete medium (containing 10% FBS) in a 37°C incubator for 3 days after which T cells were harvested and stained with anti-CD8 and anti-Thy1.1 Abs. CFSE dilution of Thy1.1^+^CD8^+^ cells was analyzed using a FACS Canto flow cytometer.

### Statistical Analysis

Data were analyzed using GraphPad Prism 5 software (GraphPad, La Jolla, CA). The Student’s *t* test was used to compare differences between pairs of samples and groups. For comparison of 3 or more groups, one-way ANOVA was used. The differences were considered statistically significant when p value was below 0.05. In figures, * indicates p < 0.05; ** p < 0.01; *** p < 0.001; **** p < 0.0001.

## Results

### The kinetics of Cell Titer Glo (CTG) luminescent assay using NIT-1 as target cells

**Figure 1:**
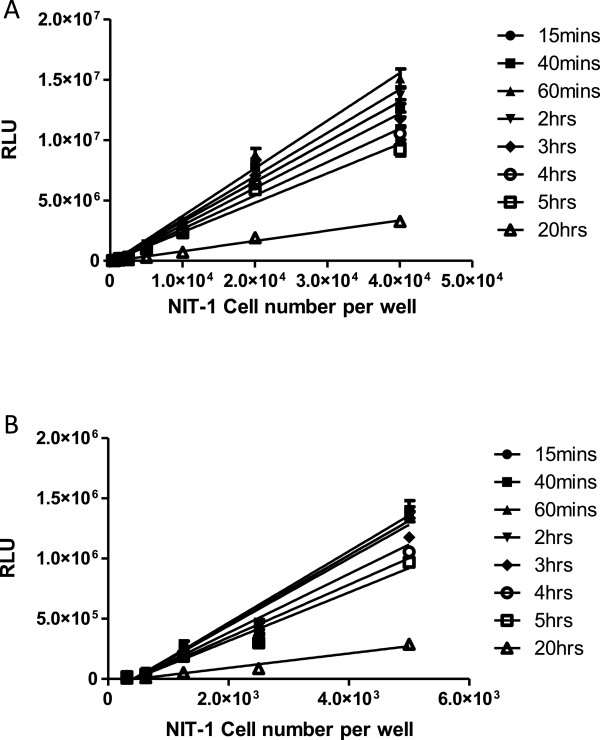
**Variation of signals in CTG method for different numbers of NIT-1 target cells and different incubation times. (A)** NIT-1 cells were cultured in RPMI medium until 80-90% confluent and then detached with cell dissociation buffer to count for use in the assay. Cells at an initial density of 4 x 10^4^ cells/well (50 μl) were titrated in 2-fold steps in a 100 μl volume per testing well. After overnight culture, the cells were washed, CTG reagent was added, and relative luminescence units (RLU) were measured at the various time points **(A)**. The same procedure was used with lower NIT-1 cell numbers **(B)**. The data shown are the mean and SD of triplicates and the results are representative of three independent experiments. For panel A, R^2^ is >0.98 for all curves (p < 0.0001); for panel B, R^2^ is >0.96 for all curves (p < 0.0001).

The CTG assay is based on the Luciferase reaction. The measurement of ATP through the luminescent signals was used to reflect the number of viable cells. As shown in Figure 1, when cell densities from 4x10^4^ cells/well down to 250 cells/well were used, the luminescent signal could be detected, and there is a good linear correlation between the RLU and the number of NIT-1 cells (R^2^ > 0.96, p < 0.0001). Following the addition of CTG reagent, the incubation time could vary from 15 min to 5 hours, but signal at 20 hours dropped significantly. Therefore, 30 minutes to 1 hour was used most of the time in the process of assay development, but the CTG incubation time could be up to 3 hours occasionally if there were a large number of samples.

### CTG assay can be used to detect killing of NIT-1 islet cells by Ag-specific 8.3 T cells

We chose to use an adherent islet cell line (NIT-1) as target cells. These cells can present the endogenously processed islet antigen IGRP _206–214_ peptide bound to K^d^ molecules, thus are recognized and lysed by 8.3 CD8^+^ T cells which bear the specific TCR recognizing this peptide: MHC class I complex.

**Figure 2:**
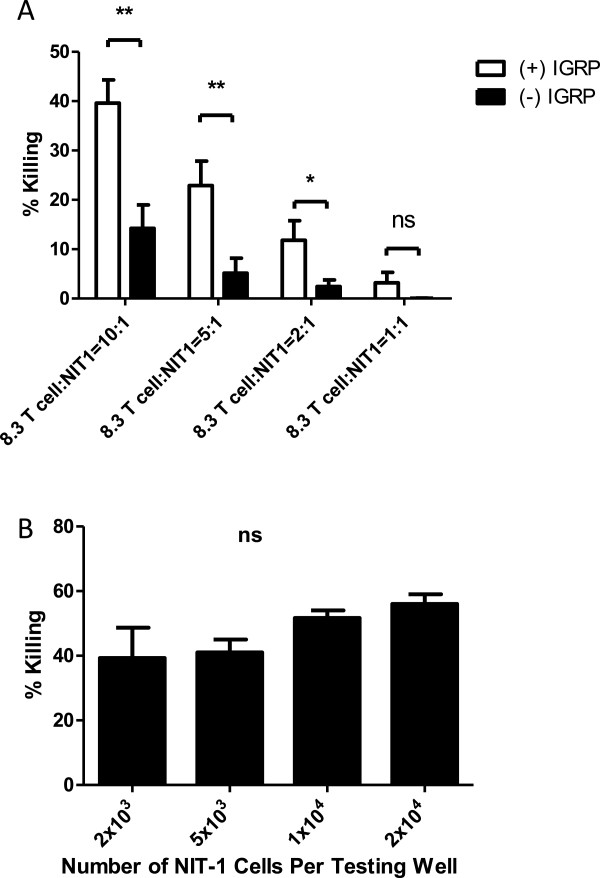
**Killing of NIT-1 islet cells by islet antigen specific 8.3 CD8** ^**+**^ **T cells in the absence or presence of exogenous IGRP peptide. (A)** The killing capacity of in vitro-activated IGRP-specific CD8^+^ 8.3 T cells was tested. NIT-1 cells or NIT-1 cells plus IGRP peptide at 20ug/ml (final concentration) were used as target cells. NIT-1 cells at the density of 2x10^3^ cells/well (50ul), equal to 4x10^4^ cells/ml, were prepared initially. Effector/Target cell (CD8 8.3 T cells/NIT-1) ratios of 10/1, 5/1, 2/1, and 1/1 were used in a final volume of 200 μl per well. After overnight incubation, 8.3 T cells were removed by washing three times with cell culture medium while adherent NIT-1 cells remained in the testing wells. Subsequently, the ATP contents reflecting the NIT-1 cell remaining viable were measured by the addition of 200 μl of 50% Cell Titer Glo followed by measurement of luminescent signals on a GloMax multi-detection system (Promega). Cytotoxicity (% killing of NIT-1 cells) was calculated as described in the material and method. Shown are mean and SD of 10 replicates for each sample. *p < 0.05; **p < 0.01 (t-test). The data are the representative of three experiments. **(B)** Based on Figure 1, the numbers of NIT-1 cells were selected for testing in the cytotoxicity assay. NIT-1 cells at 2x10^3^/well, 5x10^3^/well, 1x10^4^/well, and 2x10^4^/well were tested with the E/T ratio of 5/1. After overnight incubation, 8.3 CD8^+^ T cells were removed by washing three times with cell culture medium while adherent NIT-1 cells remained in the testing wells. Subsequently, cytotoxicity (% killing of NIT-1 cells) was measured and calculated as described in the materials and methods. Shown are the mean and SD of 10 replicates for each sample. The results are the representative of three experiments.

We tested the killing capacity of in vitro-activated IGRP-specific CD8^+^ T cells (8.3 T cells). NIT-1 cells or NIT-1 cells mixed with IGRP peptides were used as target cells. As shown in Figure 2A, at an E/T ratio of 10:1 (effector 8.3 T cells to target NIT-1 cells ratio), the islet antigen (IGRP)-specific CD8^+^ 8.3 T cells killed about 15% of NIT-1 cells after the overnight (12–16 hours) incubation. With the addition of exogenous IGRP peptide, the killing was increased to 40%. Similarly, at lower E/T ratio, 8.3 CD8^+^ T cells still killed the target NIT-1 cells better when they were mixed with IGRP peptide. Therefore, we chose to use NIT-1 cells together with addition of exogenous IGRP peptide throughout our study.

We also titrated the number of NIT-1 cells to see if the target cell number per well had any impact on 8.3 killing capacity. As shown in Figure 2B, from 2x10^3^ to 2x10^4^ cells/well, at an E/T ratio of 5:1 (8.3 T cell: NIT cell), the killing capacity remained comparable, ranging around 40-60%. Also, throughout the whole study, the killing is consistently 40 ~ 70%. Occasionally, the percentage of killing was even above 80%.

### The cytotoxicity assay is antigen-specific

**Figure 3:**
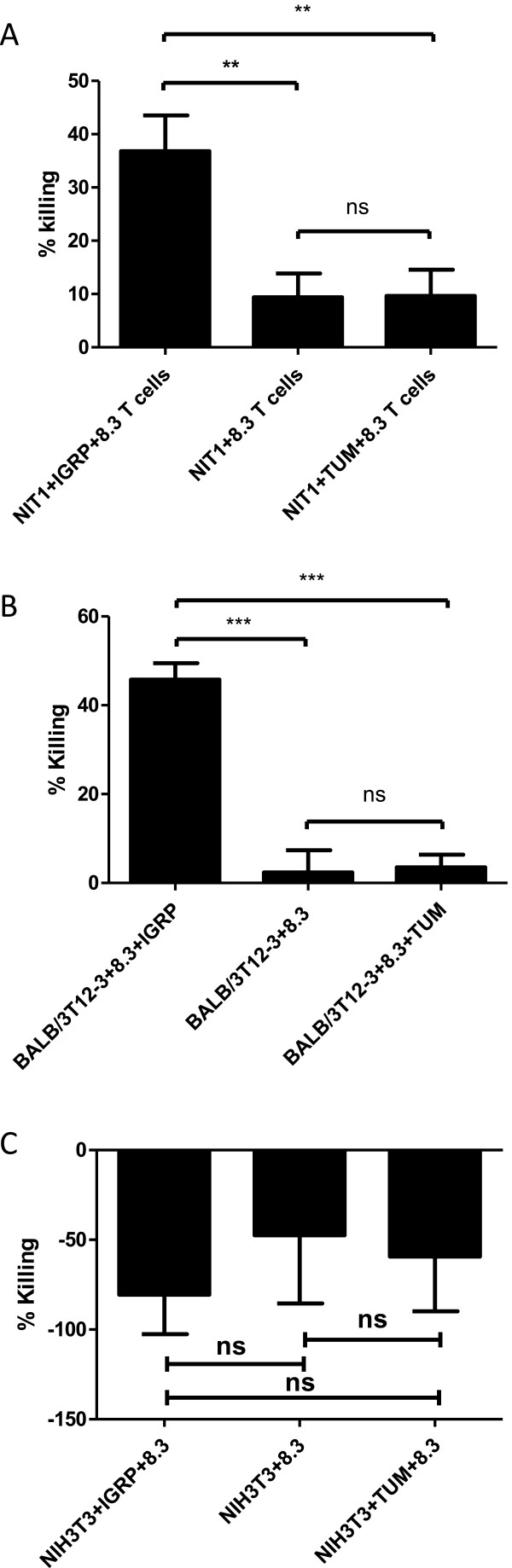
**The killing of NIT-1 islet cells was dependent on the specific recognition of the IGRP peptide/MHC I K** ^**d**^ **complex by 8.3 TCR. (A)** To verify that the cytotoxic assay is dependent on the 8.3 TCR’s specific recognition of its ligand (i.e. the IGRP peptide/K^d^ complex), a control peptide, TUM (aa sequence KYQAVTTTL) was included in parallel to IGRP; both IGRP and TUM at 20 ug/ml were tested. And NIT-1 cells at 2x10^3^/well and an E/T ratio of 5/1 were used in the assay. **(B)** BALB/3T12-3, a mouse fibroblast cell line derived from BALB/c mice and thus has the correct allele (K^d^), was also tested with the addition of exogenous IGRP peptide or the control TUM peptide. **(C)** In addition, the NIH3T3 cells, a mouse fibroblast that lacks expression of K^d^/IGRP, was tested in the same conditions as NIT1 cells. After overnight incubation, 8.3 CD8^+^ T cells were removed by washing three times with cell culture medium while the adherent NIT-1 or fibroblast cells remained in the testing wells. Subsequently, 200 μl of 50% Cell Titer Glo was added to each well followed by measurement of luminescent signals on a luminometer, and the % killing was calculated based on the RLU measured. The data shown are the representative of three experiments.

To verify the specificity of the CTG cytotoxic assay, the cytotoxic activity of 8.3 CD8^+^ T cells was examined with MHC-matched or non-matched target cells bearing the cognate peptide or an irrelevant peptide. As shown in Figure 3, although 8.3 CD8^+^ T cells were activated *in vitro* with anti-CD3/CD28 beads, if the NIT-1 cells were mixed with a control irrelevant TUM peptide (Figure 3A), the killing was the same as those without IGRP peptide (but since these are islet cells, they still have endogenous K^d^/IGRP complexes on their cell surface, thus there was still a small percentage of killing indicated by a decrease of RLU). More importantly, as a control, fibroblasts from BALB/c mice were tested as target cells, since these cells express the correct MHC class I molecule K^d^, when they were mixed with K^d^-presented IGRP peptide (the cognate peptide antigen recognized by 8.3 TCR), they could be killed by activated 8.3 T cells efficiently (~50% lysis), while the same APCs pulsed with a control irrelevant peptide (TUM) could not be killed by activated 8.3 T cells (Figure 3B). Similarly, activated 8.3 T cells did not lyse NIH3T3 cells which are of the H-2^q^ haplotype, whether these cells were mixed with IGRP peptide or not (Figure 3C). All of these data confirmed that the killing of IGRP peptide pulsed NIT-1 cells was strictly dependent on the antigen specific 8.3 TCRs which recognize the correct IGRP/K^d^ complex on the target cell surface.

### IL-2/IL-2 mAb complexes efficiently expanded endogenous Treg cells in both B6 and NOD mice

**Figure 4:**
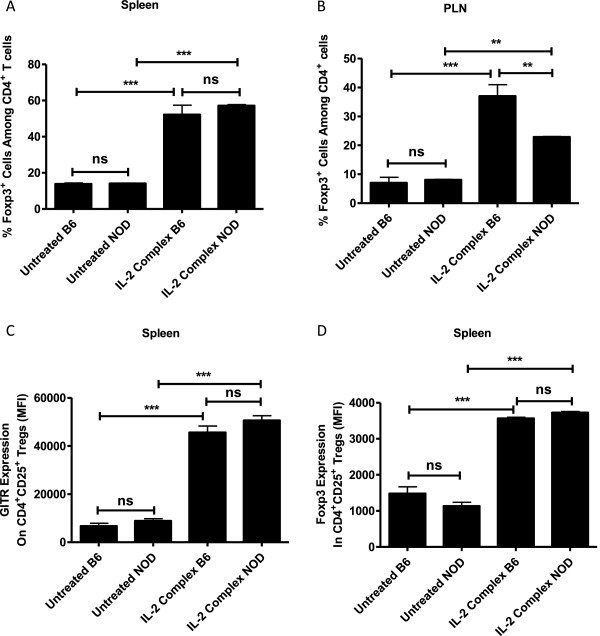
**IL-2/anti-IL-2 mAb complexes significantly expand Tregs in both B6 and NOD mice.** Female NOD mice and B6 mice (6–8 week old, n = 4/group) were left untreated or treated with IL-2/anti-IL-2mAb. On day 5 cells were isolated from lymphoid organs and labeled with anti-CD4, anti-CD8 and anti-CD25 antibodies then with anti-Foxp3 and anti-GITR antibodies and analyzed by FACS for % of Foxp3^+^ cells among CD4^+^ T cells in spleen and pancreatic lymph nodes **(A-B),** the MFI of GITR and Foxp3 on Tregs in different groups were also analyzed **(C-D).**

It has been reported that the IL-2/anti-IL-2 mAb (Clone: JES6-1) complexes can significantly expand regulatory T cells *in vivo* without affecting other subsets of immune cells [35]. We therefore administered the same IL-2/anti-IL2 mAb complexes *in vivo* in both B6 mice and NOD mice and followed the number and function of Tregs. As shown in Figure 4A and B, the administration of the IL-2/anti-IL-2 mAb complexes significantly increased the percentage of CD4^+^Foxp3^+^ Treg cells both in spleen and pancreatic lymph nodes. In the spleen, the NOD Tregs and B6 Tregs expanded to a similar degree; however, in the pancreatic lymph nodes, the expansion of B6 Tregs was significantly higher than that of NOD Tregs. When the expression of GITR and Foxp3 was analyzed (Figure 4C and D), it was clear that the IL-2 complexes treatment significantly enhanced the expression of both Treg markers, and there was no significant difference between the treated B6 and NOD group (n = 4/group).

Also, we checked the purity of freshly isolated Treg by staining with anti-CD4 and anti-CD25 antibodies and flow cytometry. As shown in Additional file 1: Figure S1A, the purity of isolated Tregs was well above 95%. And the majority of the Tregs expressed Foxp3 and GITR, two hallmark molecules that are defined as the signature markers of Tregs (Additional file 1: Figure S1B).

### Inhibitory activity of Treg cells from NOD and B6 mice at the effector phase

**Figure 5:**
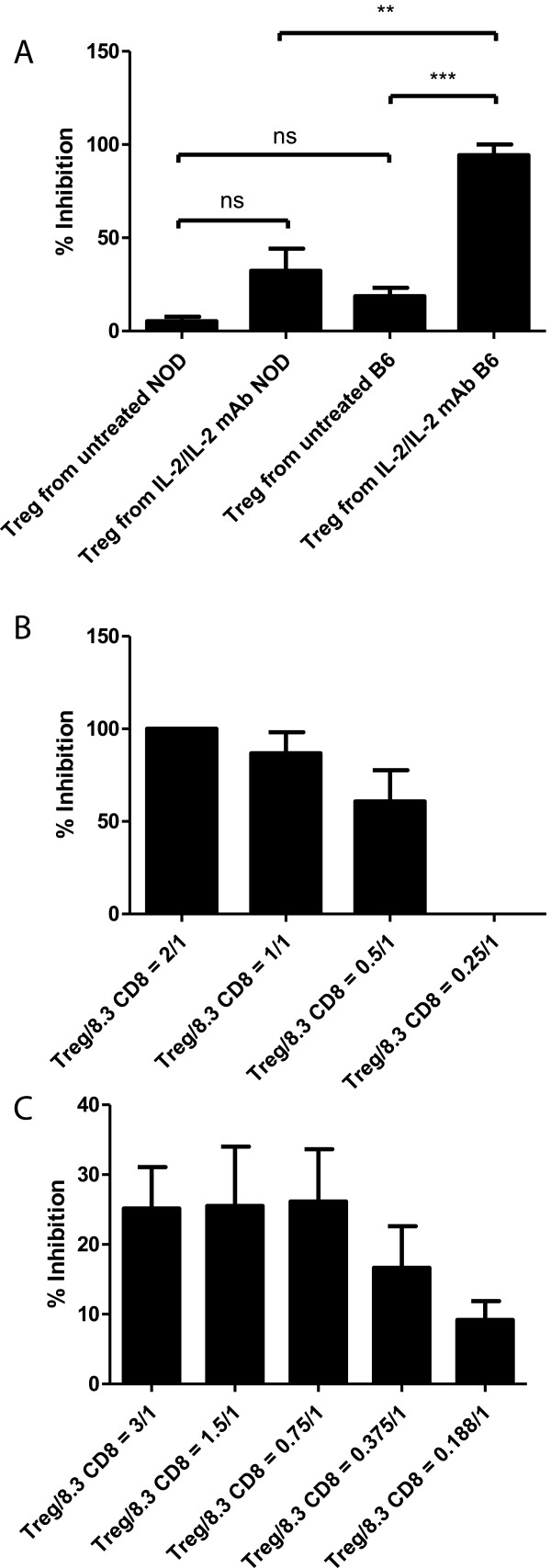
**Inhibitory activity of Treg cells from NOD and B6 mice at the effector phase. (A)** IL-2/anti-IL-2mAb complexes enhanced the immmunosuppressive activities of endogenous regulatory T-cells at the effector phase. **(B)** Impact at the effector phase of varied numbers of Treg cells isolated from B6 mice treated with IL-2/anti-IL-2mAb complexes. **(C)** Impact at the effector phase of varied numbers of Treg cells isolated from NOD treated with IL-2/IL-2mAb complex. 8.3 CD8 T cells were isolated from TCR transgenic 8.3 mice using the CD8^+^ T cell isolation kit from Miltenyi. They were then *in vitro* activated for 3 days with CD3/CD28 Dynabeads and human IL-2. On the day of the assay, Treg cells from mice untreated or treated with IL-2/anti-IL-2mAb complexes for 3 days (i.p) were isolated using the CD4^+^CD25^+^ Treg isolation kit from Miltenyi. The 8.3 CD8 T cells activated in the culture were separated from Dynabeads by magnet. NIT-1 cells were plated at 2000/well (50 μl), equal to 4x10^4^ cells/ml. 8.3 CD8^+^ T cells were used at an E/T ratio of 5:1. Various concentrations of Treg were mixed with the 8.3 CD8^+^ T cells and then added to NIT-1 cells. After overnight incubation, Treg cells and 8.3 CD8^+^ T cells were removed by washing three times with cell culture medium while adherent NIT-1 cells remained in the testing wells. Subsequently, the viability of remaining NIT-1 cells was measured by the addition of 200 μl of 50% Cell Titer Glo. Mean and SD of triplicate samples were shown; and the data shown are representative of three independent experiments.

It has been reported that Treg cells from NOD mice have reduced capacity to inhibit Tconv [18]. Therefore, we measured the inhibitory activity of Treg cells from NOD mice and B6 mice at the effector phase of 8.3 CD8^+^ T cells ( i.e. the 8.3 CD8^+^ T cells had already been activated with CD3/CD28 beads and hIL-2, and had fully differentiated into cytotoxic T cells that were immediately ready to kill their target cells). 8.3 T cells were *in vitro* activated for 3 days, then mixed with Treg cells at various ratios. The mixture of 8.3 CD8^+^ T cells and Tregs was added to NIT-1 cells and incubated overnight for 12–16 hours. The suspended Treg cells and 8.3 T cells were washed away by three repeated pipetting and washing, while the adherent NIT-1 cells remained in the well. The killing of NIT-1 cells by 8.3 T cells was measured by the CTG assay. As shown in Figure 5A, Tregs from NOD mice inhibited less than 10% of 8.3 killing; while Tregs from B6 mice had inhibitory activity of about 20%. With Treg cells isolated from IL-2/anti-IL-2 mAb complexes treated NOD mice, the inhibitory activity increased to 40%. Similarly, the Tregs isolated from B6 mice treated with IL-2/anti-IL-2 mAb complexes had an inhibitory activity of more than 80%.

When Treg cells from B6 mice treated with IL-2/anti-IL-2 mAb complexes were further titrated (Figure 5B), it was clear that these Tregs had very potent inhibitory activities. They could inhibit the killing of activated effector 8.3 T cells (that had been *in vitro* activated for 3 days) by 80% at Treg to 8.3 CD8 T cell ratio of 1 to 1 and 100% at Treg to 8.3 CD8 T cell ratio of 2 to 1. At lower ratio (0.25:1 Treg to 8.3 T cells) the inhibition dropped to 0%. Figure 5C showed the titration of Treg cells from NOD mice treated with IL-2/anti-IL-2 mAb complexes, they maintained higher suppressive activity until 0.375/1 (Treg/8.3 CD8 T cell) ratio compared with untreated Tregs (Figure 5A). In contrast to Tregs, when varied numbers of CD4^+^CD25^−^ Tconv cells were used as control, there was no inhibition of killing at all ratios (Additional file 2: Figure S2).

### Inhibitory activity of Treg cells from NOD mice at activation (stimulation) phase

**Figure 6:**
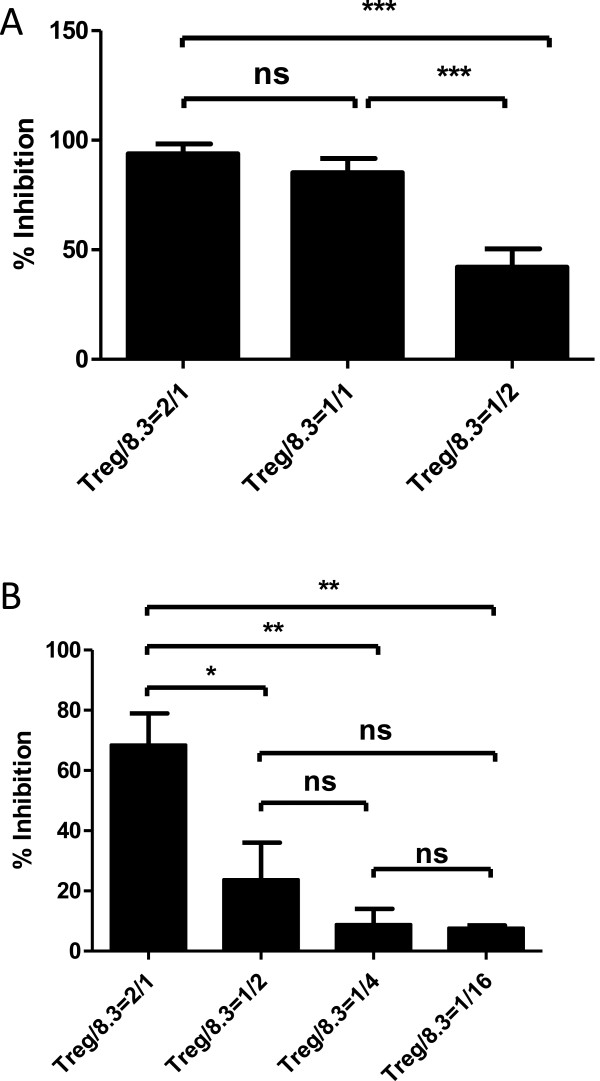
**Inhibitory activity of Treg cells from untreated and IL-2/anti-IL-2 mAb complexes treated NOD mice at the activation phase.** As described in the *materials and methods*, NOD mice were left untreated or treated with IL-2/anti-IL-2 mAb complexes. To investigate the role of Treg cells during the activation phase, freshly isolated Treg cells and 8.3 CD8^+^ T cells were cultured together in the presence of the anti-CD3/anti-CD28 Ab conjugated Dynabeads and human IL-2 for 3–4 days. On the day of assay (the last day of activation of CD8 T cells), CD8^+^ T cells were separated from Treg cells by negative selection using the mouse Dynabeads CD4 (L3T4) from Invitrogen. The NIT-1 cells were adjusted to 2000/well. The 8.3 CD8^+^ T cells were used at an E/T ratio of 5:1. The 8.3 CD8^+^ T cells and NIT-1 cells plus IGRP peptide were mixed together and incubated overnight (12 to 16 hours). After overnight incubation, 8.3 T cells were excluded by washing three times with cell culture medium while adherent NIT-1 cells remained in the testing wells. Subsequently, ATP contents reflecting the NIT-1 cell remaining viable was measured by the addition of 200 μl of 50% Cell Titer Glo. The data shown are representative of three experiments. **(A)** Tregs from untreated NOD mice. **(B)** Similar to A, but Tregs were isolated from IL-2/anti-IL-2 mAb complexes treated NOD mice. Mean and SD of triplicate samples were shown; and the data shown are representative of three independent experiments.

We next wanted to investigate whether 8.3 T cells’ killing capacity can be inhibited more efficiently if the Treg cells were present during the activation phase (stimulation phase) of 8.3 CD8^+^ T cells (i.e. during the 3-day initial activation period when naïve resting 8.3 CD8^+^ T cells were stimulated with CD3/CD28 beads and hIL-2). As expected, when naïve, unstimulated 8.3 CD8^+^ T cells were mixed with NOD Treg cells during the activation phase (*in vitro* activation with CD3/CD28 beads plus hIL-2 for 3 days), 3 days later their killing capacity was diminished by about 80% at the Treg/8.3 ratio of 1:1 (Figure 6A).

If Treg cells were isolated from NOD mice that have been treated with IL-2/anti-IL-2 mAb complexes, their inhibitory activity during the activation phase did not improve dramatically (Figure 6B). We speculate that Tregs may have a maximal suppressive activity; IL-2/anti-IL-2 mAb treatment may not be able to further improve Treg’s inhibitory activity when Tregs were already very potent during the stimulation phase. This phenomenon and its mechanism need further investigation. Also, in direct contrast to CD4^+^CD25^+^ Tregs, CD4^+^CD25^−^ Tconv cells did not significantly inhibit 8.3 killing during the activation phase (data not shown).

### IL-2/IL-2 mAb complexes efficiently enhanced the immunosuppressive ability of Tregs

**Figure 7:**
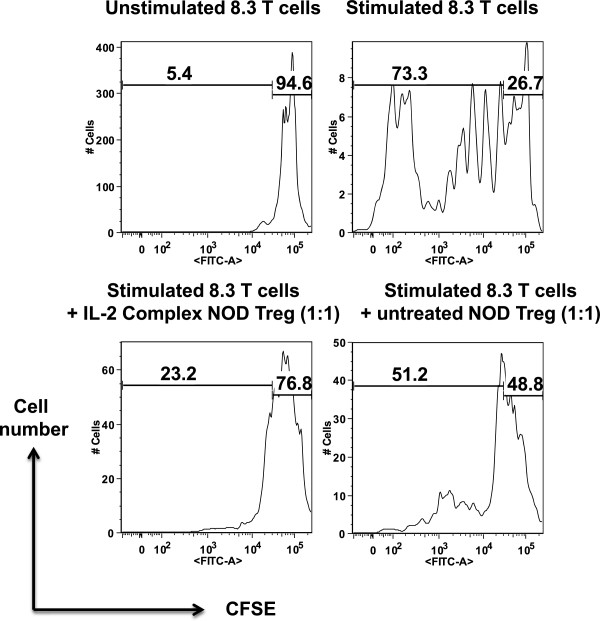
**IL-2/anti-IL-2mAb complexes enhanced the inhibitory activity of Tregs in NOD mice as measured by CFSE assay.** Female NOD mice 6–8 week old were left untreated or treated with IL-2/anti-IL-2mAb. On day 5 cells were isolated from lymphoid organs, CD4^+^CD25^+^ Tregs were purified from both groups, and co-cultured with equal number of CFSE-labelled CD8^+^Thy1.1^+^ 8,3 T cells in the presence of anti-CD3/anti-CD28 beads (2:1 beads: cell ratio). 3 days later, cells were stained with antibodies (anti-CD4, anti-CD8, anti-Thy1.1), and analyzed on a flow cytometer. The CFSE dilution (% proliferation) of 8.3 CD8^+^ T cells (gated on CD8, Thy1.1 double positive population) was plotted. Shown are the data from one representative experiment out of two independent experiments with similar results.

Besides testing the Treg’s function using the new method developed here, we were also attempting to measure the immunosuppressive activity of Tregs with the commonly used CFSE assay. As shown in Figure 7, IL-2/anti-IL-2 mAb treated NOD Tregs exhibited significantly higher inhibition of conventional T cell proliferations (in this case 8.3 CD8 T cells activated with anti-CD3/anti-CD28 beads).

### Treg cells inhibited the formation of clusters during the activation phase of 8.3 CD8^+^ T cell activation

**Figure 8:**
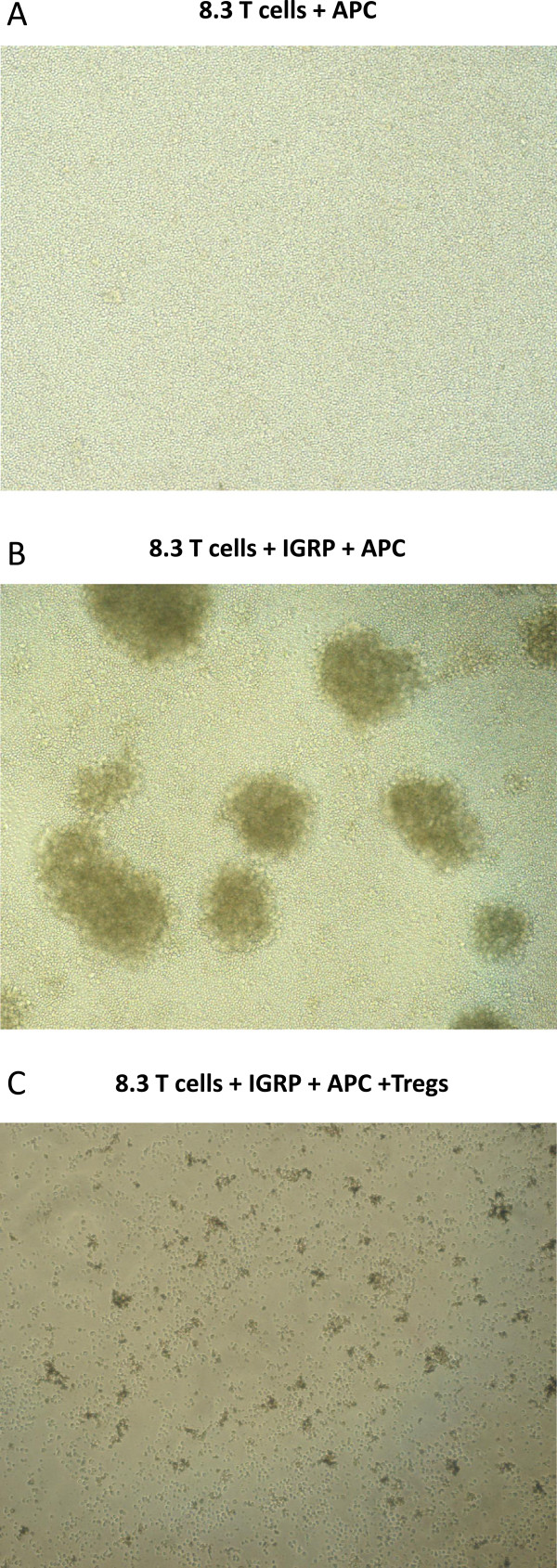
**Treg cells inhibited the formation of clusters during the activation phase of 8.3 CD8** ^**+**^ **T cell activation.** The figure shows the bright field images (100X) of CD8^+^ 8.3 T cells un-stimulated **(A)** or stimulated with specific IGRP peptide **(B),** or stimulated with specific IGRP peptide in the presence of Tregs **(C)** for 72 hours. Irradiated (3,000 Rad) splenocytes from NOD mice were used as APCs. The results are the representative of 3 different individual experiments with similar findings.

When 8.3 CD8^+^ T cells were resting, they were round and small cells under the microscope. After activation with the specific antigen-pulsed APCs, they formed large clusters, a characteristic morphology of the activated effector T cells. However, if Tregs were present from the beginning of the stimulation phase, the cluster formation almost totally disappeared (Figure 8), suggesting that Tregs suppressed the activation of resting, islet antigen-specific CD8 T cells. Additionally, we found that when 8.3 CD8^+^ T cells were activated with anti-CD3/anti-CD28 beads, the presence of Tregs also inhibited the formation of clusters (Additional file 3: Figure S3).

## Discussion

In this study, we have developed a simple and standardized *in vitro* bioassay to detect CD4^+^CD25^+^ Treg cell-mediated suppression. The sensitive CellTiter-Glo® (CTG) method was capable of measuring the Treg cell’s capacity to inhibit CTL lysis of its specific target cells. The assay was quantitative over a broad range of cell numbers and worked at both effector and stimulation phase during CD8 T cell activation.

We showed that Treg cells from untreated NOD had minimal inhibitory activity during the effector phase. However, Treg’s activity at this stage could be enhanced by the IL-2/anti-IL-2 mAb treatment. Interestingly, Tregs from untreated NOD mice had much greater inhibitory activity at the activation phase compared to that at the effector phase of CD8 8.3 T cell differentiation. Tregs from a different strain of mice (B6) on the other hand demonstrated higher inhibitory activities than NOD mice overall. This is consistent with the fact that B6 *Idd3* allele provides T1D resistance while NOD *Idd3* confers susceptibility to T1D development [36]. It has been hypothesized that IL-2 is one of the two key candidate genes in the *Idd3* locus, because the presence of an *Idd3* susceptibility gene compared with the T1D resistant allele results in lower expression of IL-2 [37], and the lower expression of IL-2 can leads to T1D susceptibility due to impaired function of Tregs [38].

The assay can be used to measure the suppressive activity of Treg on 8.3 CD8 T cells at both effector and activation phases. Different mechanisms of immunosuppression are reported to be utilized by Tregs [39], and the mechanisms might be different during the activation phase and the effector phase of the conventional T cells. For instance, the effector T cells may be killed by Tregs through granzyme and perforin-mediated cytolysis [40, 41]; while during the activation phase, the Tregs may consume the IL-2 cytokine present in the environment and act as an IL-2 “sink” [42], or negatively regulate the interaction of Tconv with DCs [43], thus preventing a productive, complete T cell activation.

Interestingly, the cluster formation of activated 8.3 T cells was inhibited by the presence of Tregs. This could be due to the fact that Tregs constitutively express the IL-2 receptor alpha chain (CD25), thus acting as a “sink” for IL-2 that limits the activation of naïve 8.3 T cells. Another possibility is that the expression of adhesion molecules required for cluster formation following T cell activation (such as LFA-1) might be negatively modulated by Tregs. All these mechanisms need to be further investigated.

Moreover, using the CTG assay as a platform, screening of chemicals such as VitD3, rapamycin, retinoic acid, HDAC (histone deacetylase) inhibitors and cytokines (IL-10, TGF-β, IL-4 etc.) and various combination of chemicals and cytokines for the most optimal conditions of Treg expansion and culture can be achieved. Even further, the *in vitro* findings can be correlated with *in vivo* models (T1D model in NOD mice, skin transplantation between allogeneic mice) to establish a preclinical model to facilitate the development of Treg-mediated immunotherapies. Since the common interest in Tregs as a candidate product to treat immune-mediated diseases has increased over time, establishing these animal models and understanding the immune regulation mechanisms may be extremely helpful in seeking new treatments for autoimmune diseases.

This new method has certain advantages over some previously established methods for evaluating Treg functions. The most commonly used technique to measure Treg activity is to label the Tregs and the responder T cells with [^3^H] thymidine and measure the incorporation. There are a number of disadvantages with this method. A major shortcoming is the radioactive isotope usages. Second, the assay usually needs more responder cells and Tregs (usually from 5x10^4^ to 5x10^5^ cells). Third, the labeling typically requires up to 18 hours of incubation time. Another approach to evaluate Treg function is CFSE labeling. This method requires larger number of cells to be labeled (up to several millions). Since Tregs only constitute 5 to 10% of CD4^+^ T cells in peripheral blood, an assay that needs lower number of Tregs will be ideal. In this study, we defined the broad range of target cell numbers from several hundred up to 4x10^4^ per testing well. And we only needed 10^4^ Tregs/well for the assay. On the other hand, there were published examples of the CFSE assay being performed with fewer cells in a 96 well plate format (such as using 2.5x10^4^-10^5^ cells per well) [22]. Even in this case, the advantage of the CTG assay is that it can measure the suppression of effector function rather than only proliferation.

Although in this study we used NIT-1 cells and 8.3 T cells as target and effector cells, this method can be easily adapted and modified depending on what effector cells and target cells are available. Other researchers can establish their own system without NIT-1 cell or 8.3 T cells. However, it should be noted that non-adherent cells as target cells cannot be used in this method.

In this study, islet-antigen specific CD8 T cells were used as the responder T cells, and antigen-specific lysis of islet cells was used as the functional readout to be inhibited by Treg cells. This has particular relevance for Treg-related research focused on tolerance induction and prevention of diabetes without the use of immunosuppressive drugs, which themselves can have severe long-term side effects, such as toxicity to the transplanted islet cells, and increased susceptibility to infections or cancers. The system could use human islet antigen-specific T cells as responder cells, and is a versatile platform for other studies if other self-tissue antigen specific T cells (such as MBP-specific T cells, insulin-specific T cells, thyroid antigen-specific T cells) are used as responder T cells.

In sum, self-tissue antigen-specific clonal T cells can be used to measure, characterize, and quantify the *in vitro* immunosuppressive activity of regulatory T cells, representing a promising approach to evaluate the various aspects of Tregs in mediating immunosuppression.

## Electronic supplementary material

Additional file 1: Figure S1: Confirmation of the purity and surface marker expression of purified Treg cells. FACS was performed on freshly isolated CD4^+^CD25^+^ Treg samples or CD4^+^CD25^−^ Tconv cells from untreated NOD mice to evaluate their purity. (A) Expression of CD4 and CD25 on the gated CD4^+^ cells. (B) Expression of Foxp3 and GITR on the gated CD4^+^CD25^+^ Tregs. The results are the representative of three experiments. (PPT 294 KB)

Additional file 2: Figure S2: Tconv did not inhibit the killing of NIT-1 by activated 8.3 CD8^+^ T cells. 8.3 CD8 T cells were isolated, *in vitro* activated for 3 days with CD3/CD28 Dynabeads and human IL-2. On the day of the assay, CD4^+^CD25^−^ conventional T cells (Tconv) from mice treated with IL-2/anti-IL-2mAb complexes for 3 days (i.p) were isolated using the CD4^+^CD25^+^ Treg isolation kit from Miltenyi (take the CD4^+^CD25^−^ fraction that was not bound to the column). 8.3 CD8^+^ T cells were used at an E/T ratio of 5:1. Various concentrations of Tconv were mixed with the 8.3 CD8^+^ T cells and then added to NIT-1 cells. After overnight incubation, cytotoxicity (% killing of NIT-1 cells) was measured as described in the materials and methods. Mean and SD of 5 replicates for each sample were shown. (PPT 100 KB)

Additional file 3: Figure S3: Treg cells inhibited the formation of clusters during the activation phase of 8.3 CD8^+^ T cell stimulated with CD3/CD28 beads. The figure shows the bright field images (100X) of CD8^+^ 8.3 T cells stimulated with CD3/CD28 beads for 72 hours in the absence (A) or presence of Tregs (1:1 Treg/8.3 ratio) from untreated NOD mice (B). The results are the representative of 3 different individual experiments with similar findings. (PPT 3 MB)

## Abbreviations

Treg: Regulatory T cell
TCR: T cell receptor
MFI: Mean fluorescence intensity
MHC: Major histocompatibility complex
APC: Antigen-presenting cell
Foxp3: Forkhead box P3
T1D: Type 1 diabetes
IL: Interleukin
IFN: Interferon
TGF-β: Transforming growth factor-beta
NOD: Non-obese diabetic
E/T ratio: Effector/target cell ratio
RLU: Relative luminescent unit
CFSE: Carboxyfluoroscein diacetate, succinimidyl ester.
