# Supplementary material for: Evaluation of immunosuppressive function of regulatory T cells using a novel in vitro cytotoxicity assay
Source: Cell Biosci. 2014 Sep 1;4:51. doi: 10.1186/2045-3701-4-51 (PMC4407464; doi:10.1186/2045-3701-4-51)
Supplement: Supplementary file 1 — Additional file 1: Figure S1: Confirmation of the purity and surface marker expression of purified Treg cells. FACS was performed on freshly isolated CD4+CD25+ Treg samples or CD4+CD25− Tconv cells from untreated NOD mice to evaluate their purity. (A) Expression of CD4 and CD25 on the gated CD4+ cells. (B) Expression of Foxp3 and GITR on the gated CD4+CD25+ Tregs. The results are the representative of three experiments. (PPT 294 KB) [file 13578_2014_200_MOESM1_ESM.ppt]

## Slide 1
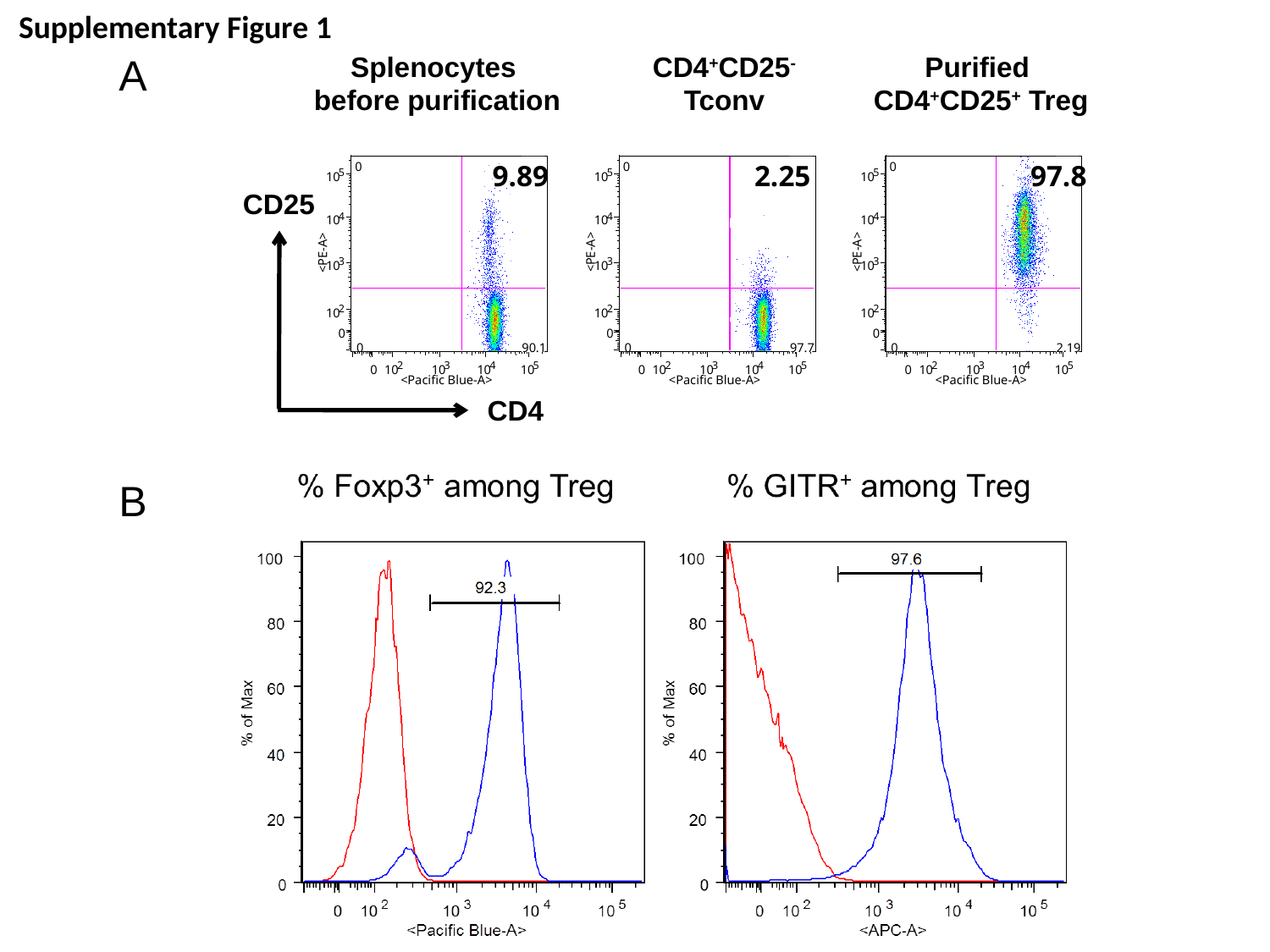

Supplementary Figure 1
A
Splenocytes
before purification
CD4+CD25-
Tconv
Purified
CD4+CD25+ Treg
5
10
4
10
3
10
2
10
0
<PE-A>
2
3
4
5
0
10
10
10
10
<Pacific Blue-A>
9.89
0
0
90.1
5
10
4
10
3
10
2
10
0
<PE-A>
2
3
4
5
0
10
10
10
10
<Pacific Blue-A>
2.25
0
0
97.7
5
10
4
10
3
10
2
10
0
<PE-A>
2
3
4
5
0
10
10
10
10
<Pacific Blue-A>
97.8
0
0
2.19
CD4
CD25
B
