# Supplementary figures and images for: Evaluation of immunosuppressive function of regulatory T cells using a novel in vitro cytotoxicity assay
Source: Cell Biosci. 2014 Sep 1;4:51. doi: 10.1186/2045-3701-4-51 (PMC4407464; doi:10.1186/2045-3701-4-51)

## Slide 1
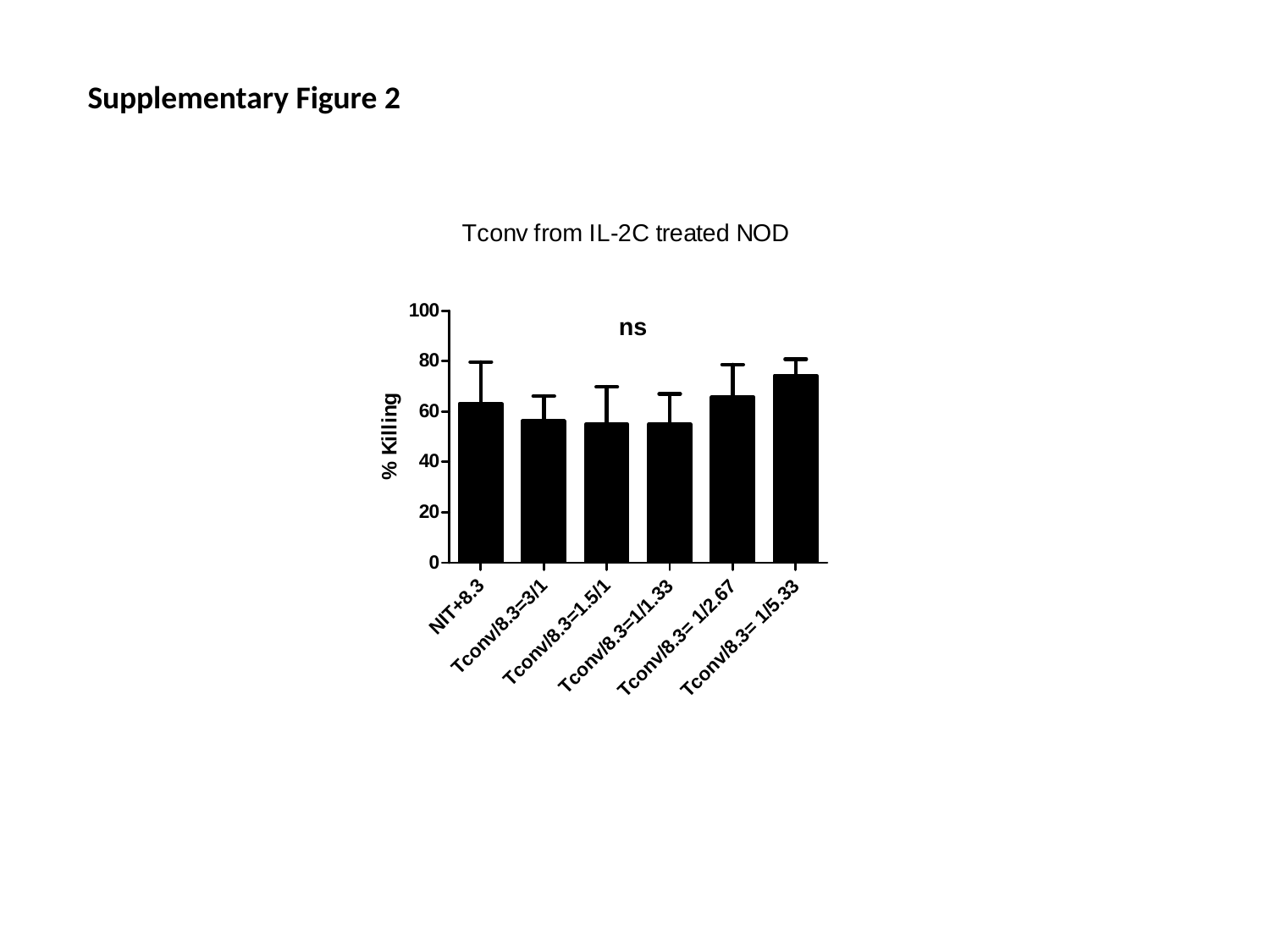

Supplementary Figure 2

Supplement: Supplementary file 2 — Additional file 2: Figure S2: Tconv did not inhibit the killing of NIT-1 by activated 8.3 CD8+ T cells. 8.3 CD8 T cells were isolated, in vitro activated for 3 days with CD3/CD28 Dynabeads and human IL-2. On the day of the assay, CD4+CD25− conventional T cells (Tconv) from mice treated with IL-2/anti-IL-2mAb complexes for 3 days (i.p) were isolated using the CD4+CD25+ Treg isolation kit from Miltenyi (take the CD4+CD25− fraction that was not bound to the column). 8.3 CD8+ T cells were used at an E/T ratio of 5:1. Various concentrations of Tconv were mixed with the 8.3 CD8+ T cells and then added to NIT-1 cells. After overnight incubation, cytotoxicity (% killing of NIT-1 cells) was measured as described in the materials and methods. Mean and SD of 5 replicates for each sample were shown. (PPT 100 KB) [file 13578_2014_200_MOESM2_ESM.ppt]
