# Supplementary material for: Evaluation of immunosuppressive function of regulatory T cells using a novel in vitro cytotoxicity assay
Source: Cell Biosci. 2014 Sep 1;4:51. doi: 10.1186/2045-3701-4-51 (PMC4407464; doi:10.1186/2045-3701-4-51)
Supplement: Supplementary file 3 — Additional file 3: Figure S3: Treg cells inhibited the formation of clusters during the activation phase of 8.3 CD8+ T cell stimulated with CD3/CD28 beads. The figure shows the bright field images (100X) of CD8+ 8.3 T cells stimulated with CD3/CD28 beads for 72 hours in the absence (A) or presence of Tregs (1:1 Treg/8.3 ratio) from untreated NOD mice (B). The results are the representative of 3 different individual experiments with similar findings. (PPT 3 MB) [file 13578_2014_200_MOESM3_ESM.ppt]

## Slide 1
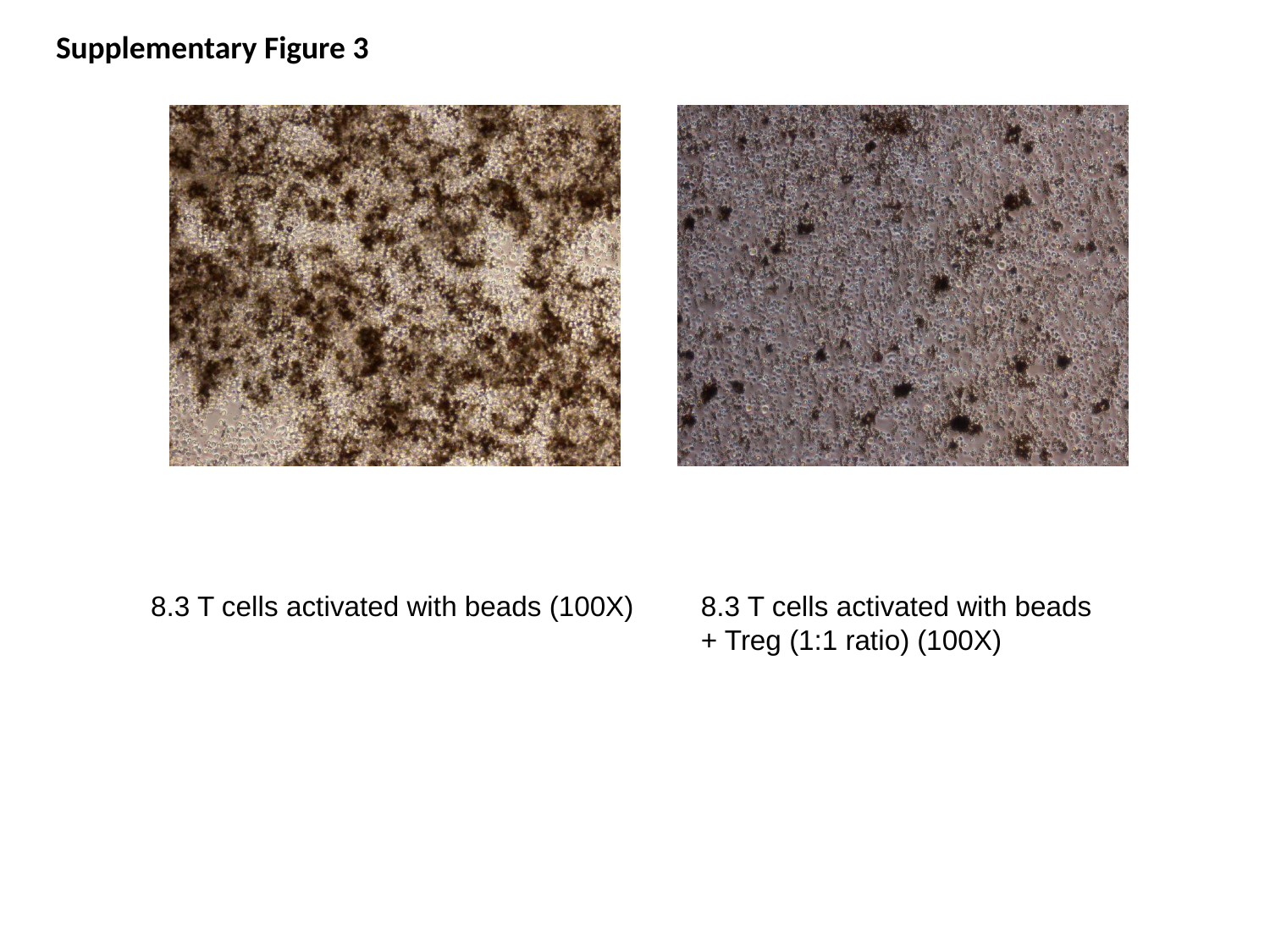

Supplementary Figure 3
8.3 T cells activated with beads (100X)
8.3 T cells activated with beads
+ Treg (1:1 ratio) (100X)
